# Population Structure of the Bacterial Pathogen *Xylella fastidiosa* among Street Trees in Washington D.C.

**DOI:** 10.1371/journal.pone.0121297

**Published:** 2015-03-27

**Authors:** Jordan Lee Harris, Yilmaz Balci

**Affiliations:** Department of Plant Science and Landscape Architecture, University of Maryland, College Park, Maryland, United States of America; Virginia Tech, UNITED STATES

## Abstract

Bacterial leaf scorch, associated with the bacterial pathogen *Xylella fastidiosa*, is a widely established and problematic disease of landscape ornamentals in Washington D.C. A multi-locus sequence typing analysis was performed using 10 housekeeping loci for *X*. *fastidiosa* strains in order to better understand the epidemiology of leaf scorch disease in this municipal environment. Samples were collected from 7 different tree species located throughout the District of Columbia, consisting of 101 samples of symptomatic and asymptomatic foliage from 84 different trees. Five strains of the bacteria were identified. Consistent with prior data, these strains were host specific, with only one strain associated with members of the red oak family, one strain associated with American elm, one strain associated with American sycamore, and two strains associated with mulberry. Strains found for asymptomatic foliage were the same as strains from the symptomatic foliage on individual trees. Cross transmission of the strains was not observed at sites with multiple species of infected trees within an approx. 25 m radius of one another. *X*. *fastidiosa* strain specificity observed for each genus of tree suggests a highly specialized host-pathogen relationship.

## Introduction

*Xylella fastidiosa* [1] is a bacterial pathogen and causal agent of numerous diseases of agricultural crops throughout the Americas. Several of the most significant diseases caused by *X*. *fastidiosa* include the Pierce’s disease of grapevine [2], citrus variegated chlorosis [3], almond leaf scald [4], and phony peach disease [5]. In addition to leaf-scorching diseases inflicted on many woody perennial cash crops, urban forests of several eastern-Atlantic municipalities share a similar fate [6–11]. In urban environments, *X*. *fastidiosa* causes a chronic leaf-scorching disease often referred to as bacterial leaf scorch (BLS) [12]. Leaf scorch symptoms first develop on an isolated tree branch and an annual progression of leaf scorch ensues, eventually leading to outright tree mortality.

*X*. *fastidiosa* is responsible for the decline of mature oak (*Quercus* spp.), elm (*Ulmus* spp.), sycamore (*Platanus* spp.), maple (*Acer* spp.), and red mulberry (*Morus rubra*) [13–15]. In New Jersey, disease incidence as high as 30% was noted for northern red oak (*Q*. *rubra*), pin oak (*Q*. *palustris*), and scarlet oak (*Q*. *coccinea*) [16]. Diseases associated with *X*. *fastidiosa* are also prevalent in Washington D.C. (the District), where a significant association was found between crown dieback and BLS infection on several common street trees including red oak, pin oak, American sycamore (*P*. *occidentalis*) and American elm (*U*. *americana*) [17]. Despite the efforts of municipal resource managers, trees begin a slow and irreversible decline once infected by *X*. *fastidiosa*. Consequently, BLS was declared an urban forest disease of concern in the 2010 Forest Action Plan for the District (http://www.forestactionplans.org/states/district-columbia).

Since the initial description of *X*. *fastidiosa* [1], four subspecies have been described, and two subspecies have been proposed. The described subspecies include i) *X*. *fastidiosa* subsp. *fastidiosa* [18], ii) *X*. *fastidiosa* subsp. *multiplex* [18], iii) *X*. *fastidiosa* subsp. *pauca* [18], iv) *X*. *fastidiosa* subsp. *sandyi* [19], while v) *X*. *fastidiosa* subsp. *tashke* [20] and iv) *X*. *fastidiosa* subsp. *morus* [21] have been proposed after MLST definition. The first three subspecies were described based on serological and phenotypic information, DNA-DNA homology, and sequencing of the 16S-23S intergenic spacer (ITS) region [18], while the later three subspecies were described thereafter once discovering separate monophyletic clade formations when constructing phylogenies using multi-locus sequence data [19,20]. Differences in host range are evident not only among these six subspecies [18,19,21,22], but also within particular subgroups, as different genotypes within a subspecies also demonstrate host specificity [23].

In several agricultural systems, the dynamics of host specificity for particular subspecific clonal complexes was demonstrated with multi-locus sequence typing (MLST), a method widely used for genetic typing and detecting recombination of *X*. *fastidiosa*. For example, *X*. *fastidiosa* subsp. *fastidiosa* is known for causing Pierce’s disease (PD) of grapevine and subsp. *multiplex* is known for causing almond leaf scorch. Both subspecies are capable of inducing almond leaf scorch [24], although subsp. *multiplex* does not induce disease of grapevine [24,25]. This anomaly of non-reciprocal symptom development after cross-inoculation was also evident in a MLST based phylogeny. While the almond leaf scorch strains were nested within both the subsp. *fastidiosa* clade and the subsp. *multiplex* clade, all PD strains were confined to the subsp. *fastidiosa* clade [26]. Using isolates derived from four regions of Brazil, *X*. *fastidiosa* strains from citrus plants were not capable of sustainable colonization of coffee plants and strains isolated from coffee plants failed to develop symptoms in citrus [27]. The strains isolated from each host species exhibited phylogenetically distinct subgroups [27–30]. Similarly, clade differentiation was evident among subspecies *fastidiosa* and subspecies *sandyi* [19], two subspecies that were proven to be incapable of infecting each other’s major host plant, grapevine and oleander, respectively [31].

Our previous survey identified several subspecies of *X*. *fastidiosa* responsible for bacterial leaf scorch in the District of Columbia [17]. *X*. *fastidiosa* subsp. *multiplex* was found in association with decline of oak, sycamore, and elm. Moreover, white mulberry was found associated with subsp. *sandyi* and a subsp. *multiplex* strain different from the subsp. *multiplex* strain found on oak, sycamore, and elm. Some of this information agrees with previous findings for oak and sycamore [18,23]. However, it has been proposed that white mulberry is infected with the newly proposed *X*. *fastidiosa* subsp. *morus* [21], and that subsp. *multiplex* [18], or at least an intermediate form of subsp. *multiplex* [23], is responsible for elm leaf scorch. It is clear that in urban environments, there exists differentiation of *X*. *fastidiosa* at the subspecies level [22]. Furthermore, whether these subspecies remain confined to a single host in a system where numerous different strains of the bacteria are endemic has not yet been demonstrated.

In order to better understand the epidemiology of leaf scorch disease associated with *X*. *fastidiosa* in urban environments, a MLST scheme for *X*. *fastidiosa* was used to genetically characterize *X*. *fastidiosa* among urban trees in the District. Our objective was to determine the strain diversity of *X*. *fastidiosa* infecting various tree species and elucidate any major clonal complexes that demonstrate host specificity.

## Materials and Methods

### Selection of *X*. *fastidiosa* Infected Trees

Sites that included trees infected with *X*. *fastidiosa* were selected throughout the District (S1 Fig). Trees in our analysis were primarily property of the District Department of Transportation, Urban Forestry Administration (DDOTUFA), except two trees that were within National Park Service (NPS) property. Permission was obtained from both DDOTUFA and NPS for all sampled trees in this study. Each site included all infected trees within an approx. 25 m radius of each other. Trees were previously determined to be infected with *X*. *fastidiosa* based on the results of a survey that used an enzyme-linked immunosorbent assay (ELISA) and PCR [17]. Samples chosen from the 2012 survey and analyzed in this investigation consisted of symptomatic and asymptomatic foliage of infected trees and symptomatic and asymptomatic foliage of neighboring infected trees. A total of 101 samples from 84 urban trees at 56 different sites were used for the MLST analysis (S1 Table). The sampled tree population consisted of 7 different species, including 20 red oaks (*Quercus rubra*), 29 pin oaks (*Quercus palustris*), 2 scarlet oaks (*Quercus coccinea*), 1 willow oak (*Quercus phellos*), 17 American elms (*Ulmus americana*), 11 American sycamores (*Platanus occidentalis*), and 4 white mulberries (*Morus alba*) (Table 1). Of the 84 sampled trees, 17 were sampled from both the symptomatic and asymptomatic portion of the crown, and 14 trees were entirely asymptomatic. The remaining 53 samples were sampled from the symptomatic region of infected tree canopies.

**Table 1:** Samples collected for the MLST analysis.

Tree Species	Total # of Trees	Total # of Samples	Asymptomatic foliage	Symptomatic foliage
Elm (*Ulmus americana*)	17	20	4	16
Mulberry (*Morus alba*)	4	7	4	3
Pin Oak (*Quercus palustris*)	29	35	12	23
Red Oak (*Quercus rubra*)	20	22	6	16
Scarlet Oak (*Quercus coccinea*)	2	2	1	1
Sycamore (*Platanus occidentalis*)	11	13	3	10
Willow Oak (*Quercus phellos*)	1	2	1	1
Total	84	101	31	70

Symptomatic and asymptomatic trees infected with *X*. *fastidiosa* were sampled. Some trees were sampled from both the asymptomatic and symptomatic portion of the canopy.

### DNA extraction, Amplification and Sequencing

Total DNA was extracted from petiole samples collected from infected trees as previously described [17]. Ten housekeeping loci were selected to form the basis of MLST typing of *X*. *fastidiosa* in the District (S2 Table). These pre-established gene regions were chosen for this organism due to the consistent level of sequence type diversity among at least 5 of the loci, variety in biochemical functions, and possession of *K*_*A*_ */K*_*S*_ values typical of moderately constrained genes (< 1), *K*_*A*_ representing synonymous mutations and *K*_*S*_ representing non-synonymous mutations [19,26,32].

For all reactions, 2 μl of total DNA extract was added to 12.5 μl of 2X GoTaq Green Master Mix (Promega Corporation, Madison, WI), 1.5 μl (10 μM solution) of each primer set designed for ten housekeeping loci of *X*. *fastidiosa* (S2 Table), and 7.5 μl of molecular grade water for a total volume of 25 μl per reaction. Dilutions of DNA extracts were performed at a 1:100 ratio with nuclease free water (Promega Corporation, Madison, WI) for optimal amplification. All amplifications were performed in a BIO-RAD S1000 Thermal Cycler (BIO-RAD, Hercules, CA), and carried out with the following cycle program: 5 min at 94°C, followed by 39 cycles of for 1 min, 55°C for 1 min, and 72°C for 1 min, and a final extension step of 10 min at 72°C. The extension time was lengthened from 1 min to 90 seconds for genes greater than 1,000 base pairs. PCR products were run in a 1.5% agarose gel pre-stained with GelRed (Biotium, Hayward, CA) at a 1:10,000 ratio of stock reagent to molten agarose (Fisher Scientific, Pittsburgh, PA) and 1X sodium boric acid conductive medium [33].

Once there was visual confirmation of the target length gene product, the PCR products were cleaned using the EXOSAP-IT PCR purification kit (Affymetrix, Inc., Santa Clara, CA). When necessary, gel extractions of target length gene product were performed using the gel extraction kit Nucleospin (Macherey-Nagel Inc., Bethlethem, PA). The cleaned PCR products were sequenced with an ABI 3730XL sequencer (Applied Biosystems, Foster City, CA) by MC Lab (San Francisco, CA). Caution should be taken when collecting sequence data from DNA that is directly extracted from infected plant tissue without the isolation of the query organism. Non-specific amplification was an issue for several American elm DNA extracts and gel extraction of the target gene product was necessary. Furthermore, when using a Sanger based approach for sequencing (such as the one used for this study); the dominant strain may mask multiple co-occurring strains in an individual sample during base calling. It has been shown that when samples with DNA from PD (subsp. *fastidiosa*) and ALS (subsp. *multiplex*) are mixed together, the strain added at a higher concentration is identified during a quantitative PCR melt analysis, and that dual peaks do not occur [34].

### Sequence analysis and submission

All sequence data was processed using the software Geneious v6.1.6 [35]. The forward and reverse read of each locus were quality-trimmed, *de novo* assembled, and ambiguities were resolved with visual analysis of the chromatograph and re-sequencing when necessary. The consensus sequence was extracted from each assembly and sequences were aligned using the Geneious alignment option [35]. In order to compare the strains in our study to those published in www.pubmlst.org, an additional set of primers were required in order to meet the sequence requirements necessary for website entry. The new primers were designed to span the gene region at each locus not formerly covered by the original primers used in the analysis. The newly designed primers were created using the primer3 v0.4.0 plug-in for Geneious [36]. Alleles for each strain at each of the seven pubmlst.org MLST loci were further sequenced and included in the sample assemblies before an allelic profile of each host species was submitted to pubmlst.org (PubMLST ID: 519–525). Bacterial strains in this study were identical to previously identified strains in the Washington D.C. region on the pubmlst.org website [21,23]. All *X*. *fastidiosa* alleles found for each host species was uploaded to GenBank, accession numbers KM487213-KM487276 and KM590452-KM590457.

### Genetic Relatedness Analysis

All ten loci were concatenated for each bacterial sequence type in this analysis, denoted as (ST_10_), and compared to the pubmlst.org sequence types, denoted as (ST). A distance tree was created with all ten loci using the Geneious tree building option in Geneious [35]. A phylogenetic tree would be inappropriate given the historical intersubspecific homologous recombination (IHR) events previously determined for strains in our analysis [21]. The program was run using the Tamura-Nei genetic distance model with a neighbor-joining tree-building algorithm. Summary of the topology posteriors was visualized in a majority rule consensus tree. A bootstrap re-sampling technique was used for approximating sampling distributions. All STs_10_ were run with 5 reference sequences in order to determine relatedness of strains from the District with strains representative of pre-defined subspecies. The reference strains included in the analysis were; Temecula 1 [37], GB514 (PD) [38], and M23 [4] represented subsp. *fastidiosa*; M12 [4] represented subsp. *multiplex*; MUL0034 [39] represented the newly described subsp. *morus*, and 9a5c [40] represented the subsp. *pauca* which was used as an outgroup.

## Results

The *X*. *fastidiosa* allelic profiles from 101 samples derived from 84 infected trees yielded 5 unique STs_10_ (S3 Table). The STs_10_ in our analysis corresponded to previously established STs for *X*. *fastidiosa* published at pubmlst.org (Table 2). Each tree genus was generally associated with a single unique *X*. *fastidiosa* ST_10_; ST_10_-1 (ST-9 in pubmlst.org), associated with members of the red oak family; red oak, pin oak, scarlet oak, and willow oak; ST_10_- 2 (ST-8) associated with American sycamore, ST_10_-3 (ST-41) associated with American elm, and ST_10_-4 and ST_10_-5 (both a derivative of ST-29) associated with mulberry (Table 3). The allelic profiles of the three loci, *gltT*, *holC*, and *cysG*, were capable of distinguishing the sequence types found within each genus of host.

**Table 2:** *Xylella fastidiosa* sequence types from tree petiole samples.

Subspecies	Sequence Type	Allelic Profiles of STs in the District of Columbia									
		*holC*	*nuoL*	*gltT*	*cysG*	*petC*	*leuA*	*malF*	rfbD*	nuoN*	pilU*
*multiplex*	ST-9 (ST_10_-1)	4 (1)	3 (1)	4 (1)	5 (1)	3 (1)	3 (1)	5 (1)	(1)	(1)	(1)
*multiplex*	ST-8 (ST_10_- 2)	4 (1)	3 (1)	7 (3)	5 (1)	3 (1)	3 (1)	5 (1)	(1)	(1)	(1)
*multiplex*	ST-41 (ST_10_-3)	9 (2)	3 (1)	3 (2)	18 (2)	3 (1)	3 (1)	5 (1)	(1)	(1)	(1)
*morus*	ST-29 (ST_10_-4)	5 (3)	4 (2)	3 (2)	18 (2)	3 (1)	4 (2)	6 (2)	(1)	(2)	(2)
*morus*	ST-29 (ST_10_-5)	5 (3)	4 (2)	3 (2)	18 (2)	3 (1)	4 (2)	6 (2)	(1)	(3)	(2)

The MLST analysis determined the presence of five clonal sequence types in the District.

() Values in parenthesis correspond to alleles and sequence types in this analysis.

* asterisk denotes additional loci examined for sequence typing in this analysis.

**Table 3:** Consensus of sequence type distribution among street trees.

Sequence Types	Number of samples with ST			
	Oak	Mulberry	Sycamore	Elm
ST-9 (ST_10_-1)	62 (100%)	0	0	1 (5%)
ST-8 (ST_10_-2)	0	0	13 (100%)	0
ST-41 (ST_10_-3)	0	0	0	19 (95%)
ST-29 (ST_10_-4)	0	3 (43%)	0	0
ST-29 (ST_10_-5)	0	4 (57%)	0	0

Each sequence type was generally host specific.

**Fig 1:**
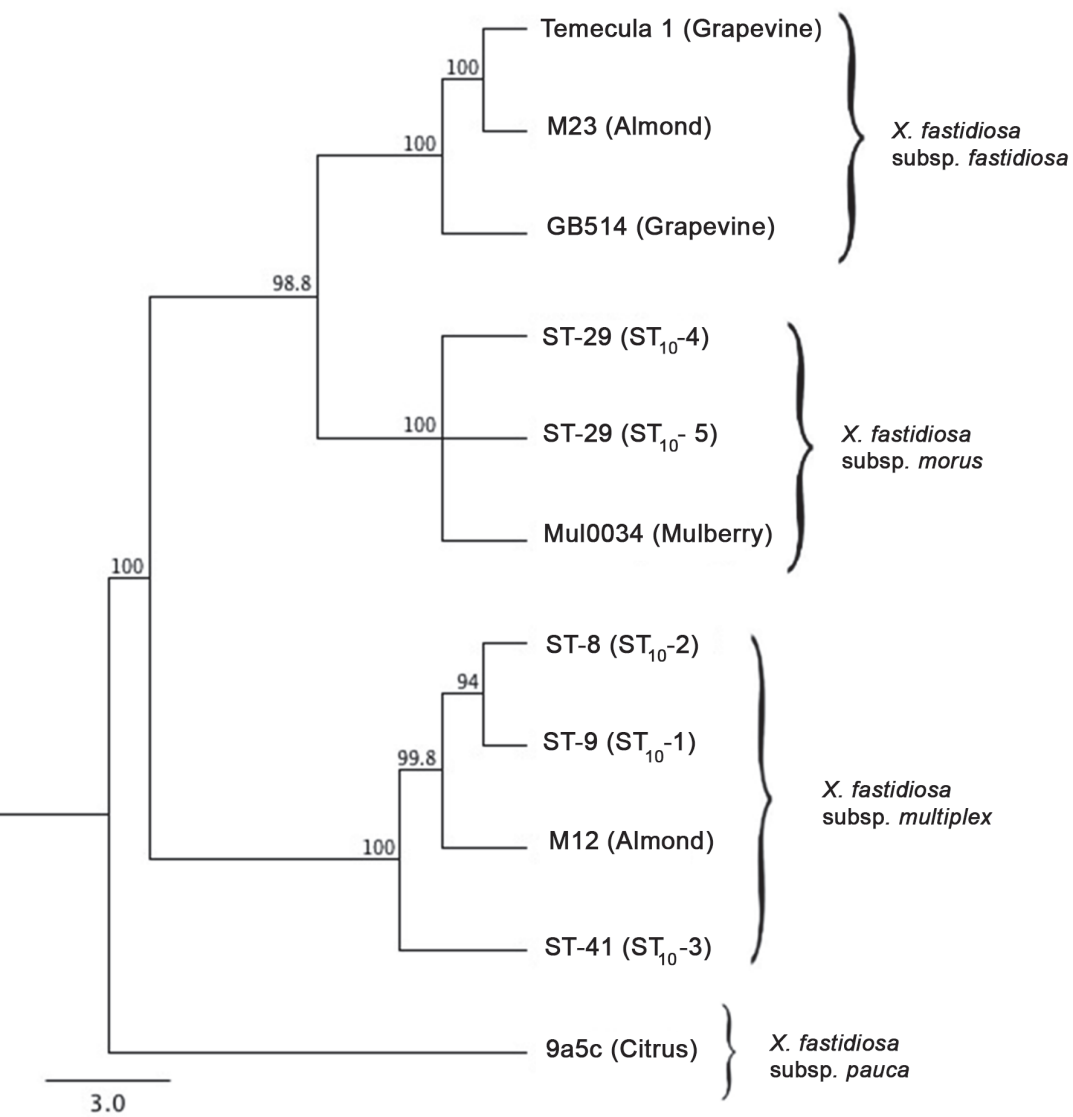
Distance tree of sequence types found infecting urban trees. A distance tree was constructed with 7,416 bp of concatenated sequence data for each *X*. *fastidiosa* sequence type. The two mulberry strains form a clade that represents the newly described subspecies *morus*, while amenity tree strains nest closely within the subsp. *multiplex* clade. Percentages represent bootstrap support from the re-sampling distribution. () Values in parenthesis represent the ten locus sequence types in this analysis.

Once the sequence types were identified, a distance tree was created using concatenated sequences of all ten loci totaling 7,416 bp for each ST_10_. Two distinct clusters were formed in the distance tree: the mulberry strains form a monophyletic group with the *X*. *fastidiosa* subsp. *fastidiosa* reference strains Temecula1, GB92.1, and M23, while the amenity tree STs_10_ were monophyletic with the subsp. *multiplex* strain M12 (Fig 1). The two clonal complexes in our analysis, defined as members within a complex sharing at least seven of ten loci, were from the mulberry strains and the amenity tree strains. The two strains from mulberry that comprise one of these complexes differed from each other by a single SNP at the *nuoN* locus (S4 Table). Within the amenity tree complex, the sycamore ST_10_-2 and oak ST_10_-1 differed from each other by a single SNP at the *gltT* locus. Elm dominant ST_10_-3 was the outlier in this complex, only sharing 7 loci with oak ST_10_-1 and sycamore ST_10_-2. The elm ST_10_-3 alleles for *gltT* and *cysG* were identical to the alleles for the mulberry ST_10_-4 and ST_10_-5 and different from the oak ST_10_-1 and sycamore ST_10_-2 strains.

STs_10_ found within the asymptomatic portions of infected trees and entirely asymptomatic trees were consistently identical to the STs_10_ found in the symptomatic canopies of the respective tree genus. The only exception occurred for a single mulberry tree that possessed both ST_10_-4 on one side of the canopy and ST_10_-5 on the other side of the canopy. Of the 56 sites selected for this study, 8 of them possessed two different tree species that were infected with *X*. *fastidiosa* and were within 25 m of each other (S1 Table). At 4 sites, infected mulberry trees were within 25 m of infected oaks, infected American elms, and infected sycamores. In 4 other sites, 1 site possessed an infected sycamore next to an infected red oak (< 25 m), 1 site possessed an infected sycamore next to an infected pin oak, 1 site possessed an infected red oak next to an infected American elm, and 1 site possessed an infected sycamore next to an infected American elm. Cross-transmission of the genus-specific STs_10_ did not occur at any of the 8 sites. The only instance where a ST_10_ was found infecting more than one genus of tree was with the oak ST_10_-1 strain found infecting one elm tree. The symptomatic elm tree was in an area that did not have a neighboring infected oak tree within 25 m.

## Discussion

The evidence of highly selective host-pathogen associations between each genus of tree and each subspecific ST is perhaps the most important observation in this study. Our results support and help explain why cross-inoculation studies of *X*. *fastidiosa* subsp. *multiplex* from different hosts have failed to demonstrate reciprocated symptom development in several instances [18,41]. This degree of host specificity was demonstrated when two strains of *X*. *fastidiosa* isolated from elm and sycamore were only pathogenic to the seedlings of their respective host plant, and cross inoculation of the isolated strains did not cause symptoms, nor could be recovered by culturing, when introduced to the reciprocate plant host [41].

Our analysis did not use cultured *X*. *fastidiosa* isolates for typing. The resulting sequence data from our analysis is only representative of the dominant sequence type in each sample. A single elm tree infected with ST-9 was the only discrepancy in host specificity observed for each ST. Although this suggests that ST-9 is capable of infecting both oaks and elms, recent inoculation by the vector, temporary persistence in the host, and presence of dual strain habitation are a few explanations that could explain why the oak strain was rarely observed infecting elm. The oak type strain ST-9 has been isolated from elm and sycamore in a previous study, which may suggest that this is a less incident but compatible association [23].

Our observation of host specificity overlaps and strongly supports the results of a former study that investigated the occurrence of *X*. *fastidiosa* throughout the southeastern United States, Texas, and California [23]. ST-9 is widespread throughout these geographic regions, and is only observed infecting oaks. In addition to ST-9, oaks have also been found associated with additional *X*. *fastidiosa* strains; ST-23 and ST-44 was found associated with Kentucky red oaks and ST-8 and ST-44 was found associated with pin oak [23]. In our analysis, the sycamore dominant strain ST-8 had previously been isolated from oak in Kentucky, but was not observed in any of the oak samples in this investigation [23]. ST-8 has also been found associated with elm [23].

It still remains unclear why specificity occurs within each of our sampling areas. Our results are counter-intuitive to proximity based strain occurrence. Despite the aforementioned evidence of compatible host overlap of ST-9 and ST-8 with oak, sycamore, and elm, each genus of tree in sites with multiple genera of infected trees possessed a specific strain of *X*. *fastidiosa*. The evidence suggests a very close host-pathogen association. Our results support the classification of the new subsp. *morus*, and ST-29 was found associated with all mulberry samples in our analysis [21]. The mulberry samples were derived from unmanaged vegetated areas, which raises the question of whether inoculum reservoirs of *X*. *fastidiosa* strains specific for amenity trees exist in such environments.

## Conclusion

Since the first documented case of elm leaf scorch in Washington D.C. [42], bacterial leaf scorch (BLS) of urban trees has continued to perpetuate uncontrolled within this urban environment. Current management strategies for suppressing disease associated with *Xylella fastidiosa* in urban environments include injections of antibiotics, application of plant growth regulators, and use of insecticides [43–46]. However, these procedures cannot cure a tree, and only slow symptom development while potentially increasing rates of disease spread, as these plants would remain pathogen sources for extended periods of time. In response to the continued degradation of the District’s urban forest, this study was undertaken in order to develop long-term management practices that mitigate the occurrence of disease. This study provides information that can assist arboreal resource mangers with selecting tree species that are not predisposed to disease from infected trees within the planting site. Our results suggest that monocultures of BLS susceptible tree species (members of the red oak family, sycamore, and elm), particularly of the same genetic variety, should be avoided when possible. In our survey, we found one particular strain to be associated with a host genus, however, it remains to be experimentally demonstrated whether host genotype is directly associated with the pathogen genotype, suggesting a heavy selection pressure, or if the specific strains found on each host genus is due to an ecological factor (i.e. vector preferences).

## Supporting Information

S1 Fig Location of sample collection sites in Washington D.C. (TIF)

S1 Table Location and site information of all trees used in the analysis. * denotes that at least two trees of the same species were sampled at the site. ** denotes that an infected tree of a different genus was within an approx. 25 m radius.
(PDF)

S2 Table Primers and gene regions selected for the multi-locus analysis. * denotes a newly designed primer for this analysis.
(PDF)

S3 Table Allelic profiles of each sample in the analysis. * denotes an outlying allelic profile from the consensus host specific sequence types. () Parenthesis indicate the allele numbers found on pubmlst.org after resequencing with additional primers.
(PDF)

S4 Table All polymorphic sites found for each allele in our analysis. Reference strains M12 (subsp. *multiplex*) and Temecula1 (subsp. *fastidiosa*) were also included for comparison.
(PDF)

S5 Table Accession numbers of unique sequence types in public database for molecular typing (Pubmlst.org). (XLS)
