# Supplementary figures and images for: Population Structure of the Bacterial Pathogen Xylella fastidiosa among Street Trees in Washington D.C
Source: PLoS One. 2015 Mar 27;10(3):e0121297. doi: 10.1371/journal.pone.0121297 (PMC4376734; doi:10.1371/journal.pone.0121297)

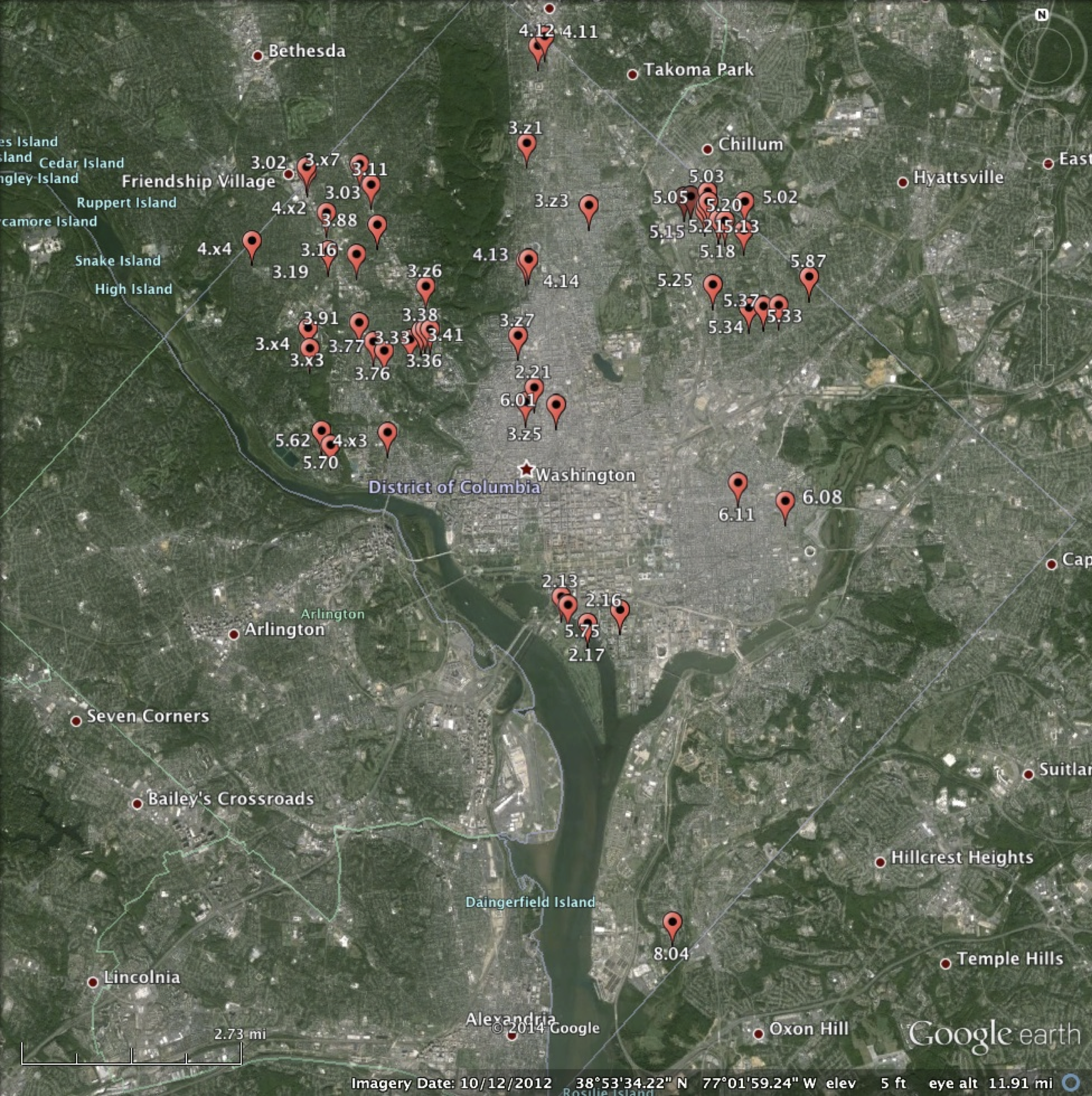

Supplement: S1 Fig — (TIF) [file pone.0121297.s001.tif]
