# Supplementary material for: Population Structure of the Bacterial Pathogen Xylella fastidiosa among Street Trees in Washington D.C
Source: PLoS One. 2015 Mar 27;10(3):e0121297. doi: 10.1371/journal.pone.0121297 (PMC4376734; doi:10.1371/journal.pone.0121297)
Supplement: S1 Table — * denotes that at least two trees of the same species were sampled at the site. ** denotes that an infected tree of a different genus was within an approx. 25 m radius. (PDF) [file pone.0121297.s002.pdf]

Table S1: Location and site information of all trees used in the analysis.

Isolate Name	Coordinates (N)	Coordinates (W)	Species	Host Common Name	Host Scientific Name	Year Collected
2.13 AS/S1 *	38° 52'44.8"	77° 00' 49.2"	*X. fastidiosa*	Pin Oak	*Quercus palustris*	2012
2.13 S1 *	38° 52'44.8"	77° 00' 49.2"	*X. fastidiosa*	Pin Oak	*Quercus palustris*	2012
2.13 S4 *	38° 52'44.8"	77° 00' 49.2"	*X. fastidiosa*	Pin Oak	*Quercus palustris*	2012
2.16 S1	38° 52'42.1"	77° 00' 45.3"	*X. fastidiosa*	Sycamore	*Platanus occidentalis*	2012
2.17 AS/S4 **	38° 52' 27.2"	77° 01' 29.3"	*X. fastidiosa*	Mulberry	*Morus alba*	2012
2.17 S1 **	38° 52' 27.2"	77° 01' 29.3"	*X. fastidiosa*	Sycamore	*Platanus occidentalis*	2012
2.17 S4 **	38° 52' 27.2"	77° 01' 29.3"	*X. fastidiosa*	Mulberry	*Morus alba*	2012
2.21 S1	38° 55' 02.8"	77° 02' 04.0"	*X. fastidiosa*	Red Oak	*Quercus rubra*	2012
3.02 AS3	38° 57' 33.9"	77° 05' 05.6"	*X. fastidiosa*	Pin Oak	*Quercus palustris*	2012
3.03 S1	38° 57' 31.0"	77° 05' 05.1"	*X. fastidiosa*	Red Oak	*Quercus rubra*	2012
3.11 AS3	38° 57' 20.3"	77° 04' 12.2"	*X. fastidiosa*	Red Oak	*Quercus rubra*	2012
3.16 S7 *	38° 56' 35.32"	77° 04' 27.65"	*X. fastidiosa*	Red Oak	*Quercus rubra*	2013
3.19 S1	38° 56' 38.6"	77° 04' 50.9"	*X. fastidiosa*	Elm	*Ulmus americana*	2012
3.33 S1	38° 55' 44.2"	77° 03' 28.9"	*X. fastidiosa*	Pin Oak	*Quercus palustris*	2012
3.36 AS3 *	38° 55' 44.7"	77° 03' 33.1"	*X. fastidiosa*	Pin Oak	*Quercus palustris*	2012
3.36 S1 *	38° 55' 44.7"	77° 03' 33.1"	*X. fastidiosa*	Pin Oak	*Quercus palustris*	2012
3.38 AS3	38° 55' 43.8"	77° 03' 36.6"	*X. fastidiosa*	Pin Oak	*Quercus palustris*	2012
3.41 S1	38° 55' 38.3"	77° 03' 46.1"	*X. fastidiosa*	Red Oak	*Quercus rubra*	2012
3.76 AS3	38° 55' 37.8"	77° 04' 16.8"	*X. fastidiosa*	Pin Oak	*Quercus palustris*	2012
3.77 AS4 *	38° 55'31.7"	77° 04' 8.2"	*X. fastidiosa*	Pin Oak	*Quercus palustris*	2012
3.77 S3 *	38° 55'31.7"	77° 04' 8.2"	*X. fastidiosa*	Pin Oak	*Quercus palustris*	2013
3.88 S1 *	38° 55'54.0"	77° 04' 08.7"	*X. fastidiosa*	Pin Oak	*Quercus palustris*	2012
3.88 S2 *	38° 55'54.0"	77° 04' 08.7"	*X. fastidiosa*	Pin Oak	*Quercus palustris*	2012
3.91 AS/S1	38° 55' 51.2"	77° 04' 28.2"	*X. fastidiosa*	Pin Oak	*Quercus palustris*	2012
3.91 S1	38° 55' 51.2"	77° 04' 28.2"	*X. fastidiosa*	Pin Oak	*Quercus palustris*	2012
3.x3 S1	38° 55' 48.83"	77° 5' 11.11"	*X. fastidiosa*	Pin Oak	*Quercus palustris*	2012

3.x4 S2	38° 55' 36.48"	77° 5' 9.996"	*X. fastidiosa*	Sycamore	*Platanus occidentalis*	2012
3.x7 S1	38° 57' 33.91"	77° 04' 21.00"	*X. fastidiosa*	Red Oak	*Quercus rubra*	2012
3.z1 AS/S5 *	38° 57' 42.56"	77° 1' 59.96"	*X. fastidiosa*	Pin Oak	*Quercus palustris*	2012
3.z1 S1 *	38° 57' 42.56"	77° 1' 59.96"	*X. fastidiosa*	Pin Oak	*Quercus palustris*	2012
3.z1 S2 *	38° 57' 42.56"	77° 1' 59.96"	*X. fastidiosa*	Pin Oak	*Quercus palustris*	2012
3.z1 S3 *	38° 57' 42.56"	77° 1' 59.96"	*X. fastidiosa*	Pin Oak	*Quercus palustris*	2012
3.z1 S5 *	38° 57' 42.56"	77° 1' 59.96"	*X. fastidiosa*	Pin Oak	*Quercus palustris*	2012
3.z1 S8 *	38° 57' 42.56"	77° 1' 59.96"	*X. fastidiosa*	Pin Oak	*Quercus palustris*	2012
3.z3 S1	38° 57' 00.33"	77° 01' 09.90"	*X. fastidiosa*	Red Oak	*Quercus rubra*	2012
3.z5 S1	38° 54' 53.71"	77° 2' 11.90"	*X. fastidiosa*	Elm	*Ulmus americana*	2012
3.z6 S1	38° 56' 12.48"	77° 3' 30.72"	*X. fastidiosa*	Elm	*Ulmus americana*	2012
3.z7 S1 *	38° 55' 37.35"	77° 2' 15.50"	*X. fastidiosa*	Pin Oak	*Quercus palustris*	2012
3.z7 S4 *	38° 55' 37.35"	77° 2' 15.50"	*X. fastidiosa*	Pin Oak	*Quercus palustris*	2012
4.11 AS/S1	38° 58' 51.9"	77° 01' 40.1"	*X. fastidiosa*	Pin Oak	*Quercus palustris*	2012
4.11 S1	38° 58' 51.9"	77° 01' 40.1"	*X. fastidiosa*	Pin Oak	*Quercus palustris*	2012
4.12 S1	38° 58' 46.1"	77° 01' 46.3"	*X. fastidiosa*	Scarlet Oak	*Quercus coccinea*	2012
4.13 S2	38° 56' 26.7"	77° 02' 03.32"	*X. fastidiosa*	Pin Oak	*Quercus palustris*	2013
4.14 S1 **	38° 56' 25.7"	77° 02' 05.2"	*X. fastidiosa*	Sycamore	*Platanus occidentalis*	2012
4.14 S2 **	38° 56' 25.7"	77° 02' 05.2"	*X. fastidiosa*	Elm	*Ulmus americana*	2012
4.98 S1	-	-	*X. fastidiosa*	Elm	*Ulmus americana*	2012
4.x2 S1	38° 57' 3.56"	77° 4' 50.73"	*X. fastidiosa*	Red Oak	*Quercus rubra*	2012
4.x3 AS/S1	38° 54' 38.28"	77°04' 9.30"	*X. fastidiosa*	Sycamore	*Platanus occidentalis*	2012
4.x3 S1	38° 54' 38.28"	77°04' 9.30"	*X. fastidiosa*	Sycamore	*Platanus occidentalis*	2012
4.x4 AS4 *	38° 56' 47.84"	77° 05' 54.59"	*X. fastidiosa*	Scarlet Oak	*Quercus coccinea*	2012
4.x4 S1 *	38° 56' 47.84"	77° 05' 54.59"	*X. fastidiosa*	Red Oak	*Quercus rubra*	2012
4.x4 S3 *	38° 56' 47.84"	77° 05' 54.59"	*X. fastidiosa*	Red Oak	*Quercus rubra*	2012
5.02 AS2 **	38° 56' 57.6"	76° 58' 58.8"	*X. fastidiosa*	Mulberry	*Morus alba*	2012
5.02 AS3 **	38° 56' 57.6"	76° 58' 58.8"	*X. fastidiosa*	Red Oak	*Quercus rubra*	2012

5.02 S1 **	38° 56' 57.6"	76° 58' 58.8"	*X. fastidiosa*	Red Oak	*Quercus rubra*	2012
5.03 S1 *	38° 57' 05.1"	76° 59' 30.0"	*X. fastidiosa*	Sycamore	*Platanus occidentalis*	2012
5.03 S2 *	38° 57' 05.1"	76° 59' 30.0"	*X. fastidiosa*	Sycamore	*Platanus occidentalis*	2012
5.05 S1	38° 56' 58.8"	76° 59' 30.5"	*X. fastidiosa*	Sycamore	*Platanus occidentalis*	2012
5.13 S1	38° 56' 56.5"	76° 59' 32.0"	*X. fastidiosa*	Pin Oak	*Quercus palustris*	2012
5.15 AS/S1	38° 56'52.6"	76° 59' 33.0"	*X. fastidiosa*	Sycamore	*Platanus occidentalis*	2012
5.15 S1	38° 56'52.6"	76° 59' 33.0"	*X. fastidiosa*	Sycamore	*Platanus occidentalis*	2012
5.18 AS2	38° 56' 46.8"	76° 59' 22.3"	*X. fastidiosa*	Red Oak	*Quercus rubra*	2012
5.2 S1 **	38° 56' 45.9"	76° 59' 16.5"	*X. fastidiosa*	Pin Oak	*Quercus palustris*	2012
5.2 S3 **	38° 56' 45.9"	76° 59' 16.5"	*X. fastidiosa*	Sycamore	*Platanus occidentalis*	2012
5.21 AS3 *	38° 56' 38.8"	76° 59' 01.3"	*X. fastidiosa*	Red Oak	*Quercus rubra*	2012
5.21 S1 *	38° 56' 38.8"	76° 59' 01.3"	*X. fastidiosa*	Red Oak	*Quercus rubra*	2012
5.25 S1	38° 56' 04.3"	76° 59' 29.6"	*X. fastidiosa*	Red Oak	*Quercus rubra*	2012
5.33 S1	38° 55' 48.1"	76° 59' 00.6"	*X. fastidiosa*	Elm	*Ulmus americana*	2012
5.34 S1	38° 55' 48.5"	76° 58' 48.3"	*X. fastidiosa*	Elm	*Ulmus americana*	2012
5.37 AS/S2 *	38° 55' 49.0"	76° 58' 35.3"	*X. fastidiosa*	Elm	*Ulmus americana*	2012
5.37 S1 *	38° 55' 49.0"	76° 58' 35.3"	*X. fastidiosa*	Elm	*Ulmus americana*	2012
5.37 S2 *	38° 55' 49.0"	76° 58' 35.3"	*X. fastidiosa*	Elm	*Ulmus americana*	2012
5.62 AS/S1 **	38° 54' 32.2"	77° 04' 57.2"	*X. fastidiosa*	Red Oak	*Quercus rubra*	2012
5.62 S1 **	38° 54' 32.2"	77° 04' 57.2"	*X. fastidiosa*	Red Oak	*Quercus rubra*	2012
5.62 S3 **	38° 54' 32.2"	77° 04' 57.2"	*X. fastidiosa*	Elm	*Ulmus americana*	2012
5.7 S2	38° 54' 41.5"	77° 05' 04.2"	*X. fastidiosa*	Elm	*Ulmus americana*	2012
5.75 AS/S2 *	38° 52' 35.1"	77° 01' 01.2"	*X. fastidiosa*	Red Oak	*Quercus rubra*	2012
5.75 S1 *	38° 52' 35.1"	77° 01' 01.2"	*X. fastidiosa*	Red Oak	*Quercus rubra*	2012
5.75 S2 *	38° 52' 35.1"	77° 01' 01.2"	*X. fastidiosa*	Red Oak	*Quercus rubra*	2012
5.82 AS/S1	38° 57' 02.1"	76° 59' 49.4"	*X. fastidiosa*	Elm	*Ulmus americana*	2012
5.82 S1	38° 57' 02.1"	76° 59' 49.4"	*X. fastidiosa*	Elm	*Ulmus americana*	2012
5.83 AS/S1	38° 57' 02.3"	76° 59' 44.5"	*X. fastidiosa*	Elm	*Ulmus americana*	2012

5.83 S1	38° 57' 02.3"	76° 59' 44.5"	*X. fastidiosa*	Elm	*Ulmus americana*	2012
5.87 AS/S1 **	38° 56' 06.6"	76° 58' 08.3"	*X. fastidiosa*	Pin Oak	*Quercus palustris*	2012
5.87 AS/S2 **	38° 56' 06.6"	76° 58' 08.3"	*X. fastidiosa*	Pin Oak	*Quercus palustris*	2012
5.87 AS/S4 **	38° 56' 06.6"	76° 58' 08.3"	*X. fastidiosa*	Mulberry	*Morus alba*	2012
5.87 S1 **	38° 56' 06.6"	76° 58' 08.3"	*X. fastidiosa*	Pin Oak	*Quercus palustris*	2012
5.87 S2 **	38° 56' 06.6"	76° 58' 08.3"	*X. fastidiosa*	Pin Oak	*Quercus palustris*	2012
5.87 S3 **	38° 56' 06.6"	76° 58' 08.3"	*X. fastidiosa*	Pin Oak	*Quercus palustris*	2012
5.87 S4 **	38° 56' 06.6"	76° 58' 08.3"	*X. fastidiosa*	Mulberry	*Morus alba*	2012
5.92 AS/S1	38° 57' 05.0"	76° 59' 29.86"	*X. fastidiosa*	Willow Oak	*Quercus phellos*	2012
5.92 S1	38° 57' 05.0"	76° 59' 29.86"	*X. fastidiosa*	Willow Oak	*Quercus phellos*	2012
6.01 S1 **	38° 54' 51.18"	77° 54' 46.42"	*X. fastidiosa*	Sycamore	*Platanus occidentalis*	2012
6.01 S2 **	38° 54' 51.18"	77° 54' 46.42"	*X. fastidiosa*	Pin Oak	*Quercus palustris*	2012
6.08 AS/S5 **	38° 53' 40.4"	76° 58' 38.2"	*X. fastidiosa*	Mulberry	*Morus alba*	2012
6.08 S1 **	38° 53' 40.4"	76° 58' 38.2"	*X. fastidiosa*	Elm	*Ulmus americana*	2012
6.08 S2 **	38° 53' 40.4"	76° 58' 38.2"	*X. fastidiosa*	Elm	*Ulmus americana*	2012
6.08 S5 **	38° 53' 40.4"	76° 58' 38.2"	*X. fastidiosa*	Mulberry	*Morus alba*	2012
6.11 S1	38° 53' 53.7"	76° 59' 18.0"	*X. fastidiosa*	Red Oak	*Quercus rubra*	2012
8.04 AS3 *	38° 49' 9.66"	77° 0' 31.36"	*X. fastidiosa*	Elm	*Ulmus americana*	2012
8.04 S1 *	38° 49' 9.66"	77° 0' 31.36"	*X. fastidiosa*	Elm	*Ulmus americana*	2012

* denotes that at least two trees of the same species were sampled at the site

** denotes that an infected tree of a different genus was within an approx. 25 m radius
