# Supplementary material for: Population Structure of the Bacterial Pathogen Xylella fastidiosa among Street Trees in Washington D.C
Source: PLoS One. 2015 Mar 27;10(3):e0121297. doi: 10.1371/journal.pone.0121297 (PMC4376734; doi:10.1371/journal.pone.0121297)
Supplement: S2 Table — * denotes a newly designed primer for this analysis. (PDF) [file pone.0121297.s003.pdf]

Table S2: Primers and gene regions selected for the multi-locus analysis.

Temecula Annotation	Temecula Coordinates	bp	Gene	Product After Translation	Biochemical function	Primer sequences	Primer References
PD0104	133,972-134,394	379	*holC*	DNA polymerase III	Replication	5'-ATGGCACGCGCCGACTTCT-3'<br>5'-ATGTCGTGTTTGTTCATGTGCAGG-3'	Yuan et al. 2010
PD0210	267,392-267,877	429	*rfbD*	dTDP-4-dehydrorhamnose-3,5-epimerase	Surface polysaccharides	5'-TTTGGTGATTGAGCCGAGGGT-3'<br>5'-CCATAAACGGCCGCTTTC-3'	Scally et al. 2005
PD0259	328,700-329,299	557	*nuoL*	NADH-ubiquinone oxidoreductase, NQO12 subunit	Aerobic respiration	5'-TAGCGACTTACGGTTACTGGGC-3'<br>5'-ACCACCGATCCACAACGCAT-3'	Yuan et al. 2010
PD0261	331,091-332,489	1311	*nuoN*	NADH-ubiquinone oxidoreductase, NQO14 subunit	Aerobic respiration	5'-GGGTTAAACATTGCCGATCT-3'<br>5'-CGGGTTCCAAAGGATTCCTAA-3'	Scally et al. 2005
PD1516	1,762,821-1,763,817	951	*gltT*	Glutamate symport protein	Transport of amonio acids	5'-TTGGGTGTGGGTACGTTGCTG-3'<br>5'-CGCTGCCTCGTAAACCGTTGT-3'	Scally et al. 2005
PD1840	2,155,901-2,157,154	1170	*cysG*	Siroheme synthase	Biosynthesis of heme, propyrin	5'-GGCGGCGGTAAGGTTG-3'<br>5'-GCGTATGTCTGTGCGGTGTGC-3'	Scally et al. 2005
PD1775	2,066,208-2,066,780	531	*petC*	Ubiquinol cytochrome c oxidoreductase, c1 subunit	Electron transport	5'-CTGCCATTCGTTGAAGTACCT-3'<br>5'-CGTCCTCCCAATAAGCCT-3'	Scally et al. 2005
PD0148	1,345,360-1,346,277	873	*pilU*	Twitching motility protein	Surface structures	5'-CAATGAAGATTCACGGCAATA-3'<br>5'-ATAGTTAATGGCTCCGCTATG-3'	Scally et al. 2005
PD1047	1,249,551-1,250,847	1218	*leuA*	2-Isopropylmalate synthase	Amino acid biosynthesis	5'-GGGCGTAGACATTATCGAGAC-3'<br>5'-GTATCGTTGTGGCGTACACTG-3'	Scally et al. 2005
PD1465	1,707,572-1,708,257	642	*lacF*	ABC transporter sugar permease	Transport of carbohydrates	5'-TTGCTGGTCCTGCGGTGTTG-3'<br>5'-CCTCGGGTCATCACATAAGGC-3'	Scally et al. 2005
PD1516*	1,763,328-1,763,833	504	*gltT*	Glutamate symport protein	Transport of amonio acids	5'-TTTTTCAGGGGTGTCGCGC-3' *<br>5'-TTCCAACGTTACTGGACGCT-3' *	This study
PD1840*	2,156,357-2,157,153	800	*cysG*	Siroheme synthase	Biosynthesis of heme, propyrin	5'-CCAAACATAGAAGCACGCCG-3' *<br>5'-CGTATGTCTGTGCGGTGTG-3' *	This study
PD1047*	1,250,469-1,250,974	564	*leuA*	2-Isopropylmalate synthase	Amino acid biosynthesis	5'-GGCCAGTGCTGTGTTTTGTT-3' *<br>5'-GGGCTACTTGCTGGAGGAAG-3' *	This study
PD1465*	1,707,432-1,708,277	829	*lacF*	ABC transporter sugar permease	Transport of carbohydrates	5'-TTCTTTGGTGGGTTGGGTGT-3' *<br>5'-CACACAGCATCAACGTCGTC-3' *	This study

* denotes a newly designed primer for this analysis
