# Supplementary material for: Population Structure of the Bacterial Pathogen Xylella fastidiosa among Street Trees in Washington D.C
Source: PLoS One. 2015 Mar 27;10(3):e0121297. doi: 10.1371/journal.pone.0121297 (PMC4376734; doi:10.1371/journal.pone.0121297)
Supplement: S3 Table — * denotes an outlying allelic profile from the consensus host specific sequence types. () Parenthesis indicate the allele numbers found on pubmlst.org after resequencing with additional primers. (PDF) [file pone.0121297.s004.pdf]

Table S3: Allelic profiles of each sample in the analysis.

Tree species	Site	Tree code	*holC*	*nuoL*	*gltT*	cysG	*petC*	*leuA*	*lacF*	*rfbD*	*nuoN*	*pilU*
Elm (*Ulmus americana*)	3.19	S1	2 (9)	-	-	-	-	-	-	-	-	-
Elm (*Ulmus americana*)	4.14	S2	2 (9)	-	2	2	-	-	-	-	-	-
Elm (*Ulmus americana*)	4.98	S1	2 (9)	-	2	-	-	-	-	-	-	-
Elm (*Ulmus americana*)	5.33	S1	2 (9)	-	2	-	-	-	-	-	-	-
Elm (*Ulmus americana*)	5.34	S1	2 (9)	1(3)	2	2	1 (3)	1	1 (5)	-	1	1
Elm (*Ulmus americana*)	5.37	S1	2 (9)	1 (3)	2 (3)	2 (18)	1 (3)	1 (3)	1 (5)	1	1	1
Elm (*Ulmus americana*)	5.37	AS/S2	2 (9)	1 (3)	2	-	1 (3)	-	1 (5)	-	1	1
Elm (*Ulmus americana*)	5.37	S2	2 (9)	1 (3)	2 (3)	2	1 (3)	1	1 (5)	-	1	1
Elm (*Ulmus americana*)	5.62	S3	2	1 (3)	2	-	1	1	1	-	1	1
Elm (*Ulmus americana*)	5.7	S2	2 (9)	-	-	-	-	-	-	-	-	-
Elm (*Ulmus americana*)	5.82	AS/S1	2	-	-	-	-	-	1	-	-	-
Elm (*Ulmus americana*)	5.82	S1	2 (9)	1 (3)	2	2	1 (3)	1	1 (5)	-	1	1
Elm (*Ulmus americana*)	5.83	AS/S1	2 (9)	-	-	-	-	1 (3)	-	-	-	-
Elm (*Ulmus americana*)	5.83	S1	2 (9)	1 (3)	2	2 (18)	1 (3)	1	1 (5)	1	1	1
Elm (*Ulmus americana*)	6.08	S1	2 (9)	-	2	-	-	-	-	-	-	-
Elm (*Ulmus americana*)	6.08	S2	2 (9)	1 (3)	2 (3)	2 (18)	1 (3)	1	1 (5)	-	1	1
Elm (*Ulmus americana*)	8.04	S1	2 (9)	-	-	-	-	-	-	-	-	-
Elm (*Ulmus americana*)	8.04	AS3	2 (9)	-	2 (3)	-	-	-	-	-	-	-
Elm (*Ulmus americana*)	3.z5	S1	2 (9)	1 (3)	2	2 (18)	1	1	1 (5)	1	1	1
Elm (Ulmus americana)	***3.z6****	***S1***	***1 (4)***	***-***	***1 (4)***	***1 (5)***	***-***	***-***	***-***	***-***	***-***	***-***
Mulberry (Morus alba)	2.17	AS/S4	3 (5)	2 (4)	2	2 (18)	1 (3)	2	2	1	3	2
Mulberry (Morus alba)	2.17	S4	3 (5)	2 (4)	2 (3)	2 (18)	1 (3)	2	2	1	2	2
Mulberry (Morus alba)	5.02	AS2	3 (5)	2 (4)	2 (3)	2 (18)	1 (3)	2	2	1	3	2
Mulberry (Morus alba)	5.87	AS/S4	3 (5)	2 (4)	2	2 (18)	1 (3)	2 (4)	2 (6)	1	3	2
Mulberry (Morus alba)	5.87	S4	3 (5)	2 (4)	2	2	1 (3)	2 (4)	2 (6)	1	3	2
Mulberry (Morus alba)	6.08	AS/S5	3 (5)	2 (4)	2	2 (18)	1	2 (4)	2 (6)	1	2	2
Mulberry (Morus alba)	6.08	S5	3 (5)	2 (4)	2	2 (18)	1 (3)	2	2	1	2	2
Pin Oak (*Quercus palustris*)	2.13	AS/S1	1 (4)	1 (3)	1 (4)	1	1 (3)	1	1	1	1	1
Pin Oak (*Quercus palustris*)	2.13	S1	1 (4)	1 (3)	1 (4)	1 (5)	1 (3)	1	1	1	1	1
Pin Oak (*Quercus palustris*)	2.13	S4	1 (4)	1 (3)	1 (4)	1	1 (3)	1	1	1	1	1
Pin Oak (*Quercus palustris*)	3.02	AS3	1 (4)	-	1	-	-	-	-	-	-	-
Pin Oak (*Quercus palustris*)	3.33	S1	1 (4)	1 (3)	1	1 (5)	1 (3)	1	1	1	1	1
Pin Oak (*Quercus palustris*)	3.36	S1	1 (4)	1 (3)	1	1 (5)	1 (3)	1	1	1	1	1
Pin Oak (*Quercus palustris*)	3.36	AS3	1 (4)	-	1	1	-	-	-	-	-	-
Pin Oak (*Quercus palustris*)	3.38	AS3	1 (4)	-	1	1 (5)	-	-	-	-	-	-
Pin Oak (*Quercus palustris*)	3.76	AS3	1 (4)	-	1	1 (5)	-	-	-	-	-	-

Pin Oak (*Quercus palustris*)	3.77	S3	1 (4)	1 (3)	1	1 (5)	1 (3)	1	1	1	1	1
Pin Oak (*Quercus palustris*)	3.77	AS4	1 (4)	-	1	1 (5)	-	-	-	-	-	-
Pin Oak (*Quercus palustris*)	3.88	S1	-	-	1	-	-	-	-	-	-	-
Pin Oak (*Quercus palustris*)	3.88	S2	1 (4)	-	1	-	-	-	-	-	-	-
Pin Oak (*Quercus palustris*)	3.91	AS/S1	1 (4)	1 (3)	1 (4)	1	1 (3)	1	1	1	1	1
Pin Oak (*Quercus palustris*)	3.91	S1	1 (4)	1 (3)	1 (4)	1 (5)	1 (3)	1	1	1	1	1
Pin Oak (*Quercus palustris*)	4.11	AS/S1	-	-	-	-	1 (3)	-	-	-	-	1
Pin Oak (*Quercus palustris*)	4.11	S1	1 (4)	1 (3)	1	1 (5)	1 (3)	1	1	1	1	1
Pin Oak (*Quercus palustris*)	4.13	S2	1 (4)	1 (3)	1	1 (5)	1 (3)	1 (3)	1 (5)	1	1	1
Pin Oak (*Quercus palustris*)	5.13	S1	1 (4)	1 (3)	1	1 (5)	1 (3)	1	1	1	1	1
Pin Oak (*Quercus palustris*)	5.2	S1	1 (4)	-	1	1 (5)	-	-	-	-	-	-
Pin Oak (*Quercus palustris*)	5.87	AS/S1	1 (4)	1 (3)	1	1	1 (3)	1	1	1	1	1
Pin Oak (*Quercus palustris*)	5.87	S1	1 (4)	1 (3)	1	1 (5)	1 (3)	1	1	1	1	1
Pin Oak (*Quercus palustris*)	5.87	AS/S2	1 (4)	1 (3)	1	1 (5)	1 (3)	1	1	1	1	1
Pin Oak (*Quercus palustris*)	5.87	S2	1 (4)	1 (3)	1	1 (5)	1 (3)	1	1	1	1	1
Pin Oak (*Quercus palustris*)	5.87	S3	1 (4)	1 (3)	1	1 (5)	1 (3)	1	1	1	1	1
Pin Oak (*Quercus palustris*)	6.01	S2	1 (4)	1 (3)	1	1 (5)	1 (3)	1	1	1	1	1
Pin Oak (*Quercus palustris*)	3.x3	S1	1 (4)	1 (3)	1	1	1 (3)	1	1	1	1	1
Pin Oak (*Quercus palustris*)	3.z1	S1	1 (4)	1 (3)	1	1 (5)	1 (3)	1	1	1	1	1
Pin Oak (*Quercus palustris*)	3.z1	S2	1 (4)	1 (3)	1	1 (5)	1 (3)	1	1	1	1	1
Pin Oak (*Quercus palustris*)	3.z1	S3	1 (4)	1 (3)	1	1 (5)	1 (3)	1	1	1	1	1
Pin Oak (*Quercus palustris*)	3.z1	AS/S5	1 (4)	1 (3)	1	1	1 (3)	1	1	1	1	1
Pin Oak (*Quercus palustris*)	3.z1	S5	1 (4)	1 (3)	1	1 (5)	1 (3)	1	1	1	1	1
Pin Oak (*Quercus palustris*)	3.z1	S8	1 (4)	1 (3)	1	1	1 (3)	1	1	1	1	1
Pin Oak (*Quercus palustris*)	3.z7	S1	1 (4)	1 (3)	1	1 (5)	1 (3)	1 (3)	1 (5)	1	1	1
Pin Oak (*Quercus palustris*)	3.z7	S4	1 (4)	-	1	1 (5)	-	-	-	-	-	-
Red Oak (*Quercus rubra*)	2.21	S1	1 (4)	1	1	-	1 (3)	1	1	-	1	1
Red Oak (*Quercus rubra*)	3.03	S1	1 (4)	-	1	1 (5)	-	-	-	-	-	-
Red Oak (*Quercus rubra*)	3.11	AS3	1 (4)	-	-	-	-	-	-	-	-	-
Red Oak (*Quercus rubra*)	3.16	S7	1 (4)	1 (3)	1	1 (5)	1 (3)	1	1	1	1	1
Red Oak (*Quercus rubra*)	3.41	S1	1 (4)	-	1	1 (5)	-	-	-	-	-	-
Red Oak (*Quercus rubra*)	5.02	S1	1 (4)	-	1	1 (5)	-	-	-	-	-	-
Red Oak (*Quercus rubra*)	5.02	AS3	1 (4)	-	1	1 (5)	-	-	-	-	-	-
Red Oak (*Quercus rubra*)	5.18	AS2	1 (4)	-	-	-	-	-	-	-	-	-
Red Oak (*Quercus rubra*)	5.21	S1	1 (4)	1 (3)	1	1	1 (3)	1	1	1	1	1
Red Oak (*Quercus rubra*)	5.21	AS3	1 (4)	-	1	-	-	-	-	-	-	-
Red Oak (*Quercus rubra*)	5.25	S1	1 (4)	1 (3)	1	1 (5)	1 (3)	1	1	1	1	1
Red Oak (*Quercus rubra*)	5.62	AS/S1	1 (4)	-	1	1 (5)	-	1	1 (5)	-	-	-
Red Oak (*Quercus rubra*)	5.62	S1	1 (4)	-	1	1 (5)	-	-	-	-	-	-

Red Oak (*Quercus rubra*)	5.75	S1	1 (4)	1 (3)	1	1 (5)	1 (3)	1 (3)	1	1	1	1
Red Oak (*Quercus rubra*)	5.75	AS/S2	1 (4)	1 (3)	1	1 (5)	1 (3)	1	1	1	1	1
Red Oak (*Quercus rubra*)	5.75	S2	1 (4)	1 (3)	1 (4)	1	1 (3)	1	1	1	1	1
Red Oak (*Quercus rubra*)	6.11	S1	1 (4)	1 (3)	1	1 (5)	1 (3)	1 (3)	1 (5)	1	1	1
Red Oak (*Quercus rubra*)	3.x7	S1	1 (4)	-	1	1 (5)	-	-	-	-	-	-
Red Oak (*Quercus rubra*)	3.z3	S1	1 (4)	-	1	1	-	-	-	-	-	-
Red Oak (*Quercus rubra*)	4.x2	S1	1 (4)	1 (3)	1 (4)	1 (5)	1 (3)	1	1	1	1	1
Red Oak (*Quercus rubra*)	4.x4	S1	1 (4)	1 (3)	1 (4)	1 (5)	1 (3)	1	1	1	1	1
Red Oak (*Quercus rubra*)	4.x4	S3	1 (4)	1 (3)	1 (4)	1 (5)	1 (3)	1	1	1	1	1
Scarlet Oak (*Quercus coccinea*)	4.12	S1	1 (4)	1 (3)	1	1 (5)	1 (3)	1	1	1	1	1
Scarlet Oak (*Quercus coccinea*)	4.x4	AS4	1 (4)	-	-	-	-	-	-	-	-	-
Sycamore (*Platanus occidentalis*)	2.16	S1	1 (4)	1 (3)	3	1 (5)	1 (3)	1 (3)	1 (5)	1	1	1
Sycamore (*Platanus occidentalis*)	2.17	S1	1 (4)	1 (3)	3	1 (5)	1 (3)	1	1	1	1	1
Sycamore (*Platanus occidentalis*)	4.14	S1	1 (4)	1 (3)	3	1 (5)	1 (3)	1	1 (5)	1	1	1
Sycamore (*Platanus occidentalis*)	5.03	S1	1 (4)	-	3	1 (5)	-	-	-	-	-	-
Sycamore (*Platanus occidentalis*)	5.03	S2	1 (4)	-	3 (7)	1 (5)	-	-	1 (5)	-	-	-
Sycamore (*Platanus occidentalis*)	5.05	S1	1 (4)	1 (3)	3	1 (5)	1 (3)	1	1	1	1	1
Sycamore (*Platanus occidentalis*)	5.15	AS/S1	1 (4)	-	3	1 (5)	-	1	1 (5)	-	-	-
Sycamore (*Platanus occidentalis*)	5.15	S1	1 (4)	1 (3)	3	1 (5)	1 (3)	1	1	1	1	1
Sycamore (*Platanus occidentalis*)	5.2	S3	1 (4)	-	3 (7)	1 (5)	-	1 (3)	-	-	-	-
Sycamore (*Platanus occidentalis*)	6.01	S1	1 (4)	1 (3)	3	1 (5)	1 (3)	1	1	1	1	1
Sycamore (*Platanus occidentalis*)	3.x4	AS/S1	1 (4)	-	-	-	-	-	-	-	-	-
Sycamore (*Platanus occidentalis*)	4.x3	AS/S1	1 (4)	-	3 (7)	1 (5)	-	1	1 (5)	-	1	1
Sycamore (*Platanus occidentalis*)	4.x3	S1	1 (4)	1 (3)	3 (7)	1 (5)	1 (3)	1	1	1	1	1
Willow Oak (*Quercus phellos*)	5.92	AS/S1	1 (4)	1 (3)	1 (4)	1 (5)	1 (3)	1	1 (5)	-	1	1
Willow Oak (*Quercus phellos*)	5.92	S1	1 (4)	1 (3)	1 (4)	1 (5)	1 (3)	1 (3)	-	-	1	1

* denotes an outlying allelic profile from the consensus host specific sequence type

( ) Parenthesis indicate the allele numbers found on pubmlst.org. Several loci required additional primers to meet the sequence requirements.
