# Supplementary material for: Population Structure of the Bacterial Pathogen Xylella fastidiosa among Street Trees in Washington D.C
Source: PLoS One. 2015 Mar 27;10(3):e0121297. doi: 10.1371/journal.pone.0121297 (PMC4376734; doi:10.1371/journal.pone.0121297)
Supplement: S4 Table — Reference strains M12 (subsp. multiplex) and Temecula1 (subsp. fastidiosa) were also included for comparison. (PDF) [file pone.0121297.s005.pdf]

**Table S4: All polymorphic sites found for the alleles in our analysis.**

Temecula Coordinates of DNA Polymorphisms

| PD0261 / *nuoN* gene | | | | | | | | | | | | | | | | |
|---|---|---|---|---|---|---|---|---|---|---|---|---|---|---|---|---|
| 1,216 bp (post-trim) | 331,327 | 331,401 | 331,524 | 331,630 | 331,639 | 331,654 | 331,774 | 331,870 | 331,888 | 332,129 | 332,131 | 332,132 | 332,242 | 332,266 | 332,287 | 332,389 |
| Allele 1 | G | G | G | T | C | C | C | G | G | C | C | T | C | T | C | T |
| Allele 2 | A | A | C | C | G | T | A | T | C | A | T | C | T | G | T | T |
| Allele 3 | A | A | C | C | G | T | A | G | C | A | T | C | T | G | T | T |
| subsp. *fastidiosa* | A | G | C | C | C | T | C | G | C | A | T | C | T | G | T | C |
| subsp. *multiplex* | G | G | G | T | C | C | C | G | G | C | C | T | C | T | C | T |

| PD0104 / *holC* gene | | | | | | | | | | | | | | |
|---|---|---|---|---|---|---|---|---|---|---|---|---|---|---|
| 360 bp (post-trim) | 134,004 | 134,053 | 134,059 | 134,089 | 134,194 | 134,266 | 134,287 | 134,289 | 134,290 | 134,293 | 134,298 | 134,299 | 134,320 | 134,367 |
| Allele 1 | T | G | A | G | G | T | C | C | G | C | G | G | T | A |
| Allele 2 | C | A | A | G | G | T | C | C | G | C | G | G | T | A |
| Allele 3 | C | A | A | G | A | C | C | T | A | T | T | A | C | A |
| subsp. *fastidiosa* | C | A | A | A | A | C | T | T | G | C | G | G | T | C |
| subsp. *multiplex* | T | G | G | G | G | T | C | C | G | C | G | G | T | A |

| PD1465 / *lacF* gene | | | | | | | | | | | | | | |
|---|---|---|---|---|---|---|---|---|---|---|---|---|---|---|
| 632 bp (post-trim) | 1,707,664 | 1,707,676 | 1,707,751 | 1,707,756 | 1,707,813 | 1,707,921 | 1,707,931 | 1,708,024 | 1,708,031 | 1,708,138 | 1,708,181 | 1,708,193 | 1,708,199 | 1,708,234 |
| Allele 1 | C | T | T | A | A | A | A | C | G | G | C | A | G | C |
| Allele 2 | C | T | G | G | G | G | T | A | A | A | G | G | G | C |
| subsp. *fastidiosa* | A | C | G | G | G | G | T | A | A | A | G | G | G | A |
| subsp. *multiplex* | C | T | T | A | A | A | A | C | G | G | C | A | C | C |

| PD0148 / *pilU* gene | | | | | | | | | | | | | | | | |
|---|---|---|---|---|---|---|---|---|---|---|---|---|---|---|---|---|
| 854 bp (post-trim) | 1,345,667 | 1,345,673 | 1,345,789 | 1,345,823 | 1,345,892 | 1,345,928 | 1,345,940 | 1,345,947 | 1,346,042 | 1,346,054 | 1,346,094 | 1,346,143 | 1,346,171 | 1,346,183 | 1,346,206 | 1,346,210 |
| Allele 1 | G | C | G | C | G | A | T | T | A | A | C | C | A | A | C | C |
| Allele 2 | T | T | A | T | A | G | C | C | G | G | G | G | C | G | A | T |
| subsp. *fastidiosa* | T | T | A | T | A | G | C | C | G | G | G | G | C | G | A | T |
| subsp. *multiplex* | G | C | G | C | G | A | T | T | A | A | C | C | A | A | C | C |

| | 1,346,240 | 1,346,241 | 1,346,243 |
|---|---|---|---|
| Allele 1 (Continued) | C | T | G |
| Allele 2 (Continued) | T | C | A |
| subsp. *fastidiosa* | T | C | A |
| subsp. *multiplex* | C | T | G |

| PD1840 / *cysG* gene | | | | | | | | | | | | | | | | |
|---|---|---|---|---|---|---|---|---|---|---|---|---|---|---|---|---|
| 905 bp (post-trim) | 2,156,101 | 2,156,122 | 2,156,417 | 2,156,474 | 2,156,484 | 2,156,509 | 2,156,614 | 2,156,721 | 2,156,728 | 2,156,869 | 2,156,896 | 2,156,944 | 2,156,951 | 2,156,958 | 2,156,977 | 2,156,987 |
| Allele 1 | A | T | C | G | T | A | G | A | C | C | T | C | G | C | C | A |
| Allele 2 | A | T | C | G | T | A | G | G | T | C | C | T | A | T | T | G |
| subsp. *fastidiosa* | G | C | T | A | C | G | G | G | T | G | C | T | A | C | T | G |
| subsp. *multiplex* | A | T | C | G | T | A | A | A | C | C | T | C | G | C | C | A |

PD1047 / *leuA* gene

| 1,119 bp (post-trim) | 1,249,732 | 1,249,875 | 1,250,004 | 1,250,127 | 1,250,412 | 1,250,637 | 1,250,639 | 1,250,665 | 1,250,667 | 1,250,685 | 1,250,688 | 1,250,748 |
|---|---|---|---|---|---|---|---|---|---|---|---|---|
| Allele 1 | G | T | C | C | C | T | A | A | T | G | T | C |
| Allele 2 | G | T | C | C | C | C | G | G | C | T | C | T |
| subsp. *fastidiosa* | A | A | G | T | T | C | G | G | T | T | C | T |
| subsp. *multiplex* | G | T | C | C | C | T | A | A | T | G | T | C |

PD0259 / *nuoL* gene

| 540 bp (post-trim) | 328,737 | 328,863 | 328,989 | 329,033 | 329,077 | 329,112 | 329,120 | 329,205 | 329,227 |
|---|---|---|---|---|---|---|---|---|---|
| Allele 1 | G | C | T | C | G | T | T | A | A |
| Allele 2 | G | C | T | C | A | T | T | G | C |
| subsp. *fastidiosa* | A | A | A | T | A | C | C | A | C |
| subsp. *multiplex* | G | C | T | C | G | T | T | A | A |

PD1516 / *gltT* gene

| 842 bp (post-trim) | 1,762,901 | 1,763,045 | 1,763,162 | 1,763,195 | 1,763,201 | 1,763,265 | 1,763,270 | 1,763,369 | 1,763,556 | 1,763,615 | 1,763,630 | 1,763,681 | 1,763,702 | 1,763,729 |
|---|---|---|---|---|---|---|---|---|---|---|---|---|---|---|
| Allele 1 | G | G | G | A | T | A | G | C | A | A | A | G | C | A |
| Allele 2 | G | G | G | A | T | G | G | C | A | G | A | G | C | A |
| Allele 3 | G | G | G | A | T | A | G | C | A | G | A | G | C | A |
| subsp. *fastidiosa* | A | A | A | G | G | G | A | G | G | G | G | A | T | T |
| subsp. *multiplex* | G | G | G | A | T | G | G | C | A | G | A | G | C | A |

PD0210 / *rfbD* gene

| 413 bp (post-trim) | 265,222 | 265,231 | 265,236 | 265,243 | 265,260 | 265,291 | 265,309 | 265,413 | 265,567 | 265,576 | 265,601 | 265,625 |
|---|---|---|---|---|---|---|---|---|---|---|---|---|
| Allele 1 | C | C | C | C | G | C | G | T | G | T | A | G |
| subsp. *fastidiosa* | T | T | T | T | A | T | T | G | G | T | A | G |
| subsp. *multiplex* | C | C | C | C | G | C | G | T | A | C | C | A |

PD1775 / *petC* gene

| 533 bp (post-trim) | 2,066,328 | 2,066,372 | 2,066,448 | 2,066,499 | 2,066,638 | 2,066,701 |
|---|---|---|---|---|---|---|
| Allele 1 | G | T | T | T | C | A |
| subsp. *fastidiosa* | A | C | C | C | G | G |
| subsp. *multiplex* | G | T | T | T | C | A |

Reference strains M12 (subsp. *multiplex*) and Temecula1 (subsp. *fastidiosa*) were also included for comparison.
